# Breast Cancer Screening in Older Adults Living with Dementia: A Scoping Review

**DOI:** 10.1093/geroni/igaf122.4078

**Published:** 2025-12-31

**Authors:** Eun-Hi Kong

**Affiliations:** Gachon University, Incheon, Republic of Korea

## Abstract

The utilization of breast cancer screening in older adults with dementia is debatable, challenging, and ethical issue. There has been a lack of evidence on breast cancer screening among older adults living with dementia. The purpose of this review to synthesize evidence of the literature regarding breast cancer screening in older adults living with dementia. Systematic literature search was conducted using electronic databases including MEDLINE, CINAHL, PsycINFO, EMBASE, and Google scholar from the earliest year to July, 2025. Search keywords included dementia, Alzheimer, cognitive impairment, cognitive impaired, cognitive dysfunction, breast cancer screening, mammography, and mammogram. Search results were uploaded and duplicates were removed using EndNote21. Both published studies and grey literature were included in this review. This study followed the PRISMA-ScR and the JBI guidance and for scoping review. Twelve studies were included in this review. Most studies were conducted in USA and used quantitative research methods. Many studies targeted older adults living with dementia and some studies targeted caregivers. The included studies had various focuses such as utilization rate, associated factor, comparison with elders without dementia, decision, benefit, harm, and cost effectiveness of breast cancer screening among older adults with dementia. Further studies are required to explore benefits, harms, decision-making, ethical issues, barriers, and facilitators of breast cancer screening among older adults living with dementia. More qualitative research and mixed methods research are needed. Future studies are needed to develop and evaluate clear and detailed guidelines on breast cancer screening in older adults living with dementia.

